# Synthesis of novel adsorbent based on tetrasulfide-functionalized fibrous silica KCC-1 for removal of Hg(II) cations

**DOI:** 10.1038/s41598-021-90279-3

**Published:** 2021-05-24

**Authors:** Azam Marjani, Reza Khan Mohammadi

**Affiliations:** Department of Chemistry, Arak Branch, Islamic Azad University, Arak, Iran

**Keywords:** Environmental social sciences, Natural hazards

## Abstract

Hg(II) has been identified to be one of the extremely toxic heavy metals because of its hazardous effects and this fact that it is even more hazardous to animals than other pollutants such as Ag, Au, Cd, Ni, Pb, Co, Cu, and Zn. Accordingly, for the first time, tetrasulfide-functionalized fibrous silica KCC-1 (TS-KCC-1) spheres were synthesized by a facile, conventional ultrasonic-assisted, sol–gel-hydrothermal preparation approach to adsorb Hg(II) from aqueous solution. Tetrasulfide groups (–S–S–S–S–) were chosen as binding sites due to the strong and effective interaction of mercury ions (Hg(II)) with sulfur atoms. Hg(II) uptake onto TS-KCC-1 in a batch system has been carried out. Isotherm and kinetic results showed a very agreed agreement with Langmuir and pseudo-first-order models, respectively, with a Langmuir maximum uptake capacity of 132.55 mg g^–1^ (volume of the solution = 20.0 mL; adsorbent dose = 5.0 mg; pH = 5.0; temperature: 198 K; contact time = 40 min; shaking speed = 180 rpm). TS-KCC-1was shown to be a promising functional nanoporous material for the uptake of Hg(II) cations from aqueous media. To the best of our knowledge, there has been no report on the uptake of toxic Hg(II) cations by tetrasulfide-functionalized KCC-1 prepared by a conventional ultrasonic-assisted sol–gel-hydrothermal synthesis method.

## Introduction

Water is the most essential and momentous component on our blue planet for marine ecosystems and vital activities of living organisms. Unfortunately, the water quality of surface and groundwater is deteriorating continuously because of increasing urbanization, industrialization, environmental changes, and agricultural activities. For all these reasons, water contamination has become a crucial problem in the present scenario, affecting humans, animals, and other organisms^1^. Thousands of biological, organic, and inorganic contaminants have been reported as water pollutants. Some of these contaminations are created by heavy metals that infected water and affected an organism’s health. Some heavy metals such as Hg(II), Pb(II), Tl(I), Tl(III), Cr(VI), and Cd(II) are notorious water contaminants with extreme toxicity, mutagenicity, and, carcinogenicity^2^. Among them, mercury is known as one of the most dangerous heavy metals, which has attracted special attention among environmental scientists. Mercury is highly carcinogenic to animals, plants, and human beings because of hindering the transport processes in living cells, and manifestations of mercury poisoning include dysfunction of the central nervous system, kidney, liver, gastrointestinal tract, and brain damage. Consequently, the removal of mercury from aqueous environments is of great importance^3,4^.

In recent decades, various techniques and strategies have been utilized for heavy metals decontamination. The most important strategies are adsorption, reverse osmosis, precipitation, ion exchange, micro-and ultra-filtration, oxidation, centrifugation, coagulation, sedimentation and gravity separation, distillation, screening, solvent extraction, evaporation, crystallization, flotation, electrodialysis, electrolysis, etc.^5–7^. Among them, the adsorption strategy is considered as one of the favorite water remediation methods owing to its ease of operation (flexibility in design and operation), high efficiency, the availability of a wide range of structures, and the ability to combine with the above-mentioned removal strategies^8–13^.

In view of the importance of the quality of water to human and animal health and emerging utilities of nanotechnology and nanomaterials, a variety of efforts have been undertaken to discover nanostructured adsorbents (nanoadsorbents) and to utilize them for water treatment by adsorption method^14–16^. Various nanostructured adsorbent substances, including graphene-based nanomaterials^17,18^, mesoporous silica materials^12,13^, zeolites^19,20^, metal/covalent organic frameworks^7,8,21^, layered double hydroxides^22,23^, and mesoporous carbons and carbon nanotubes^24–26^ have been studied over recent years. Among the aforementioned substances with the ability of adsorption, mesoporous silica materials—viz. hollow spheres, KIT-5, KIT-6, MCM-41, MCM-48, SBA-15, SBA-16, etc.—have received a great deal of interest because of their large identical pore networks, high surface area, functionalizable surface, and nontoxic nature of silica network^8,9,11,27,28^. The adsorption properties of these nanomaterials are significantly improved by functionalization (attaching metal-capturing functional groups to their surfaces). The capacity of metal binding and removing heavy metals from the environment is impacted by the limitation of target species access to surface-bounded organic ligands. A single nanoadsorbent cannot be used for all kinds of adsorbates. Accordingly, it is important to functionalized adsorbents with suitable surface-bounded organic ligands.

One of the most interesting mesoporous silica materials with high surface area is fibrous silica nanospheres (KCC-1)^29^. Recently, KCC-1 has been used in current challenges about the environment, energy, and sustainability, like sensors, adsorbents, carriers, and catalysts^11,14,28,30^. KCC-1 has a lot of accessibility on its surface because of silica fibers that exist throughout its structure, instead of pores^31^. In environmental applications, KCC-1 may help adsorb and eliminate heavy metals because of its unique properties such as fibrous silica morphology, accessible surface silanol groups with functionalization capability, high pore volume, and large surface area^11,14^.

In this work, for the first time, adsorptive removal of Hg (II) from aqueous media by a tetrasulfide-functionalized fibrous silica KCC-1 (TSF-KCC-1) was studied. Pure KCC-1 was prepared via a simple, conventional sol–gel-hydrothermal synthesis approach and then functionalized with bis[3-(triethoxysilyl)propyl] tetrasulfide (TESPTS) via a facile post-modification method. The ultrasonic-assisted preparation method was carried out because the ultrasound exposure can result in a more uniform distribution of metal capturing agents (tetrasulfide groups). Consequently, this approach facilitates the possible interactions between Hg(II) cations and tetrasulfide functional groups on the adsorbent material which is crucial to augment both kinetics of the adsorption and adsorption efficiency.

## Results and discussion

### FTIR spectroscopy

**Figure 1:**
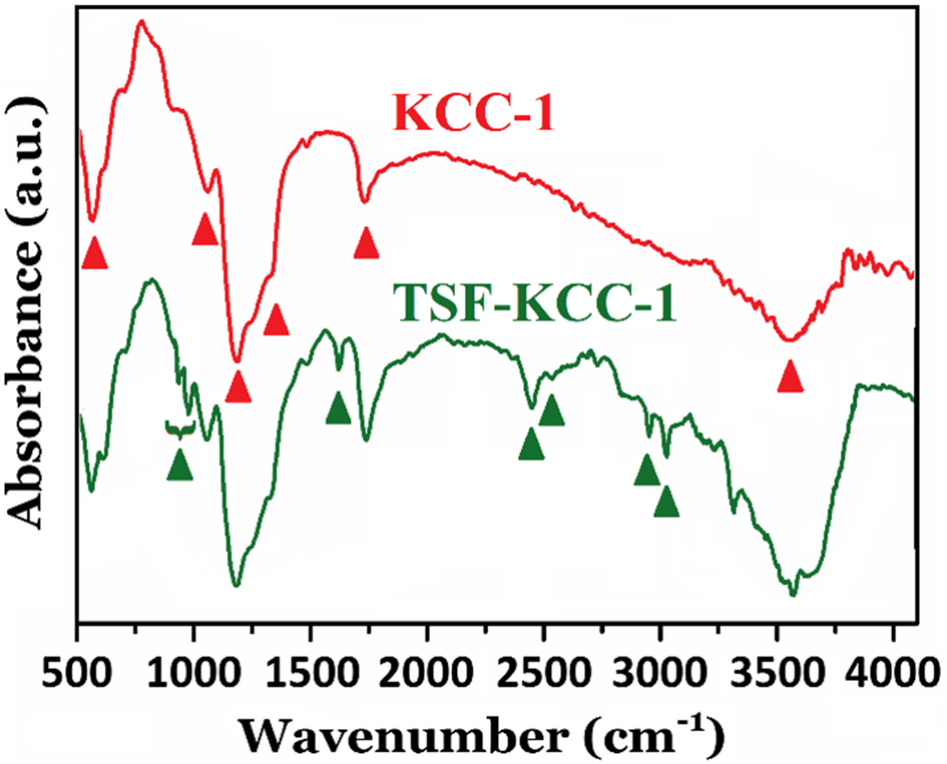
FTIR spectra of pure KCC-1 and TSF-KCC-1 materials.

FTIR spectra of the pure KCC-1 and TSF-KCC-1 were recorded from 4000 to 400 cm^–1^ and depicted in Fig. 1. KCC-1 shows a typical siliceous composition, representing FTIR bands around 3648–3000 cm^–1^, assigned to ν(SiO–H ) (vicinal, terminal, and geminal silanols) and surface-adsorbed water, at 1081 and 1210 cm^–1^, related to asymmetric ν (Si–O–Si), at 965 cm^–1^, attributed to asymmetric ν(Si–OH), around 806 cm^–1^, assigned to symmetric ν(Si–O–Si), and around 465 cm^–1^, related to bending mode of Si–O–Si^11–15,32^. For TSF-KCC-1, after modification, besides the aforementioned absorption bands, some new featured FTIR bands could be seen at around 2754 cm^–1^ 2926 cm^–1^ from ν_s_(C–H) and ν_as_(C–H) methylene groups of TESPT moiety, at around 1482 cm^–1^ (C–H bending vibration), and at 758–851 cm^–1^ (Si–C stretching mode). The abovementioned characteristic bands are absent in the FTIR spectrum of KCC-1. These changes in the FTIR spectrum of TSF-KCC-1 resulting from the successful grafting of TESPT information on the silica and the formation of the hybrid organic–inorganic structure containing.

### FESEM, TEM, and EDX mapping analyses

**Figure 2:**
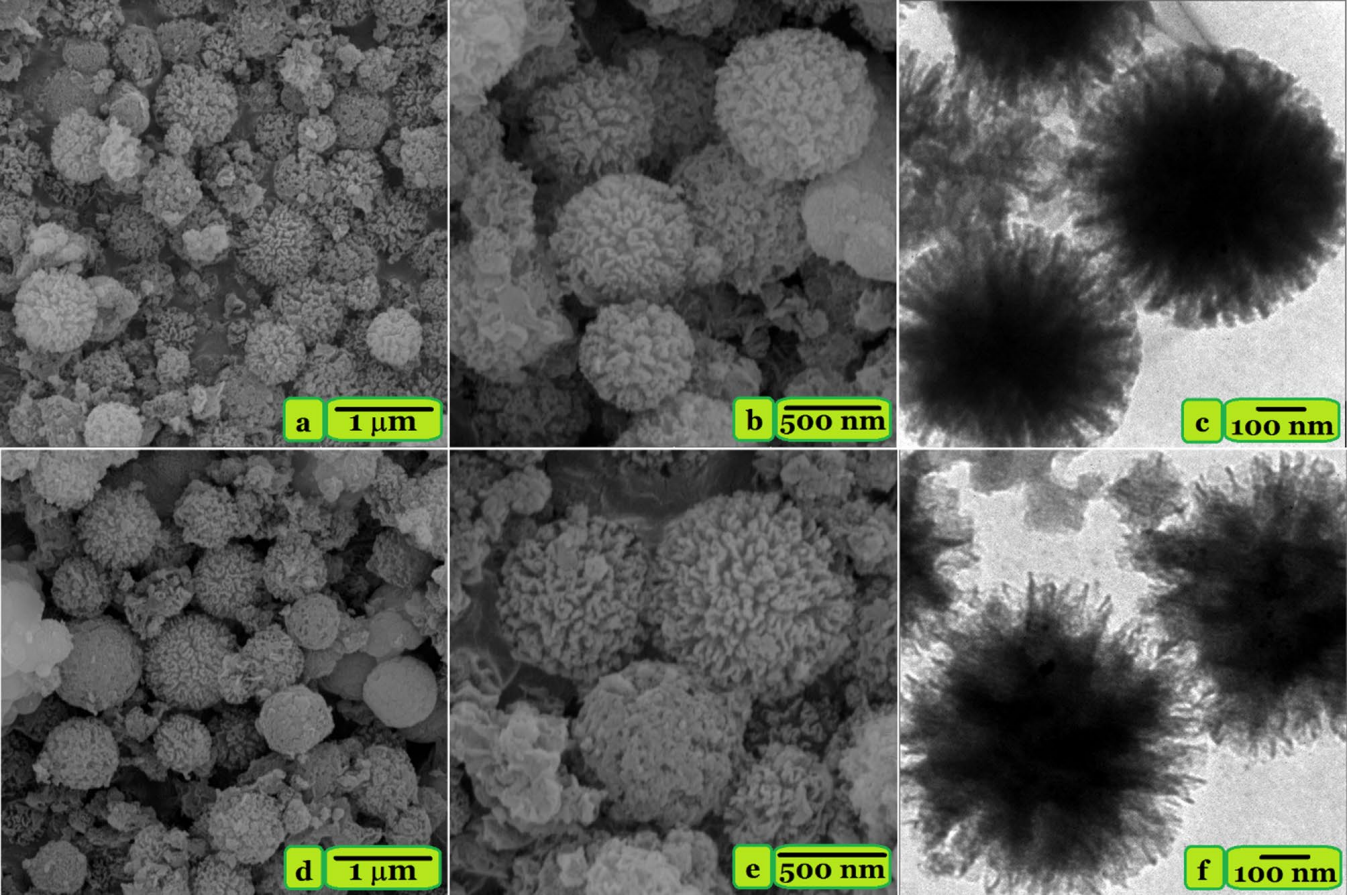
FESEM images of KCC-1 (**a**,**b**) and TSF-KCC-1 (**d**,**e**). TEM images of KCC-1 (**c**) and TSF-KCC-1 (**f**).

The FESEM and TEM micrographs of samples are shown in Fig. 2. Figure 2 a and b show a uniform spherical morphology with particle size in the range of 200 to 700 nm (submicrosphere) for pure KCC-1. After surface grafting of KCC-1 with TESPT silane coupling agent, TSF-KCC-1 shows a similar shape, demonstrating that the surface modification has no significant effect on the morphology. Furthermore, the TEM images of KCC-1 (Fig. 2c) and TSF-KCC-1 (Fig. 2f) reveal that both KCC-1 and TSF-KCC-1 spheres have a uniform fibrous silica structure. However, the silica fibers in TSF-KCC-1 are somehow irregularly arranged, while in the case of KCC-1, the fibers are distributed more regularly and uniformly in all directions. This observation has also been reported by Soltani et al. where the regularity of the fibers in the mesoporous KCC-1 is decreased upon surface functionalization with various silane coupling agents^11,14^.

**Figure 3:**
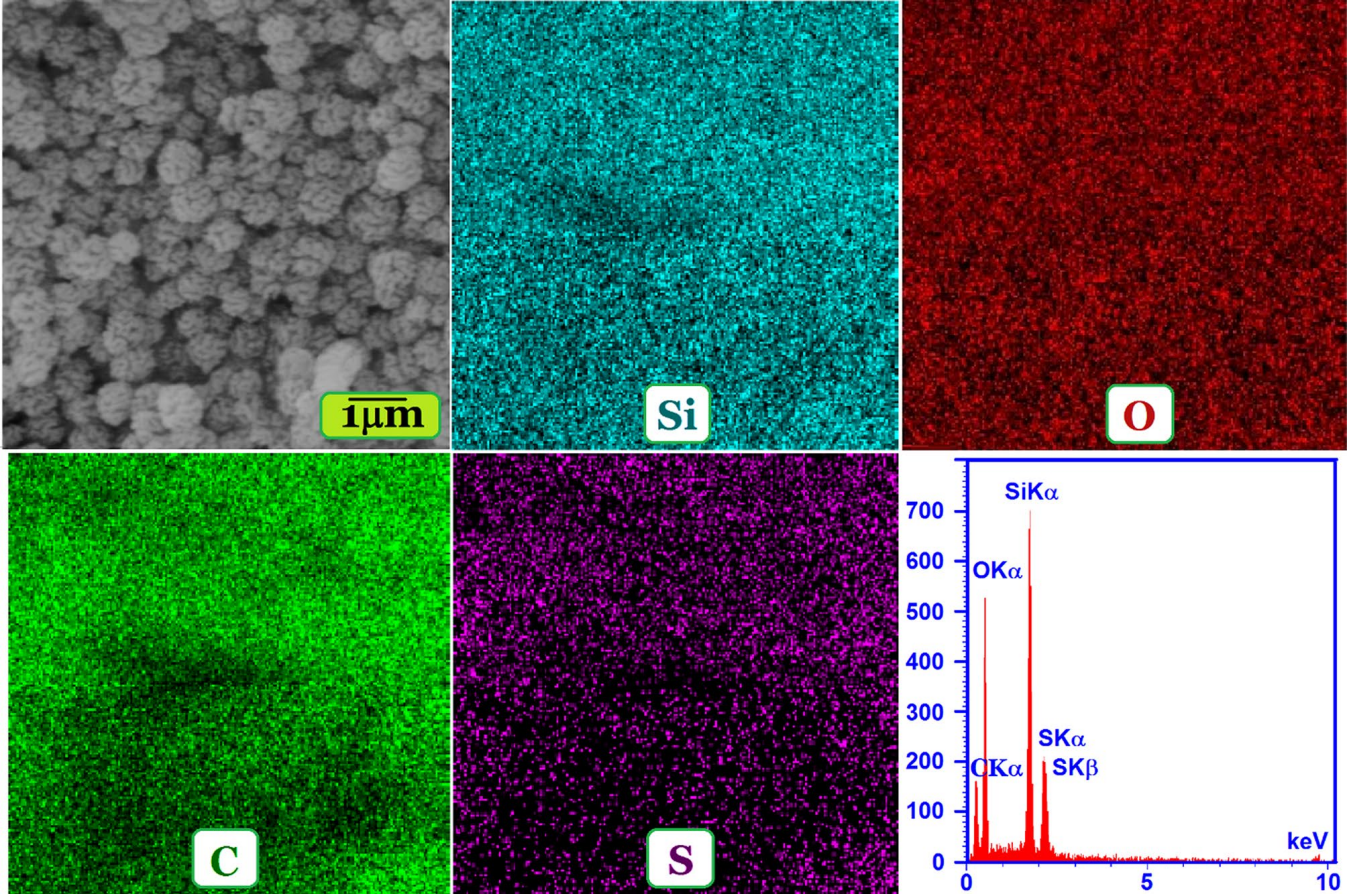
EDS and EDS elemental mapping of TSF-KCC-1 material.

EDS spectrum and EDS elemental mapping images of TSF-KCC-1 are given in Fig. 3. The elemental mapping analysis reveals a uniform distribution of silicon (Si), carbon (C), sulfur (S), and oxygen (O) elements within the whole TSF-KCC-1 submicrospheres indicating a degree of purity in the surface of the TSF-KCC-1 adsorbent. The uniform distribution of these structural elements may be owing to the fact that the ultrasonic-assisted sol–gel-hydrothermal preparation method for synthesis of TSF-KCC-1 adsorbent leads to the formation of a uniform modified surface layer. Also, the EDS spectrum revealed peaks in the Si, S, C, and O regions, confirming the successful grafting of TESPT on the surface of TSF-KCC-1 material.

**Figure 4:**
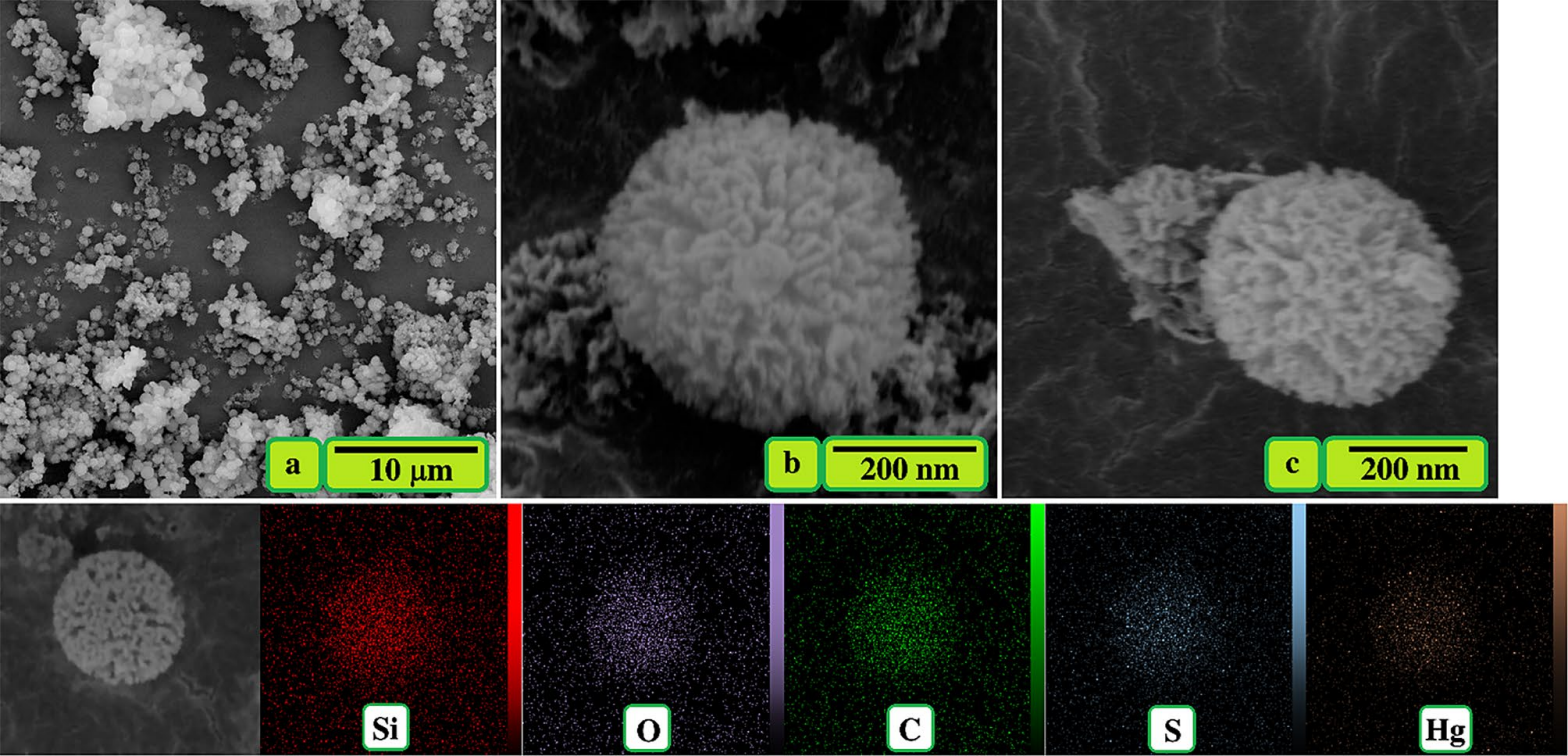
FESEM (**a**–**c**, first row) and EDS elemental mapping (second row) images of TSF-KCC-1 adsorbent after Hg(II) adsorption.

FESEM and EDS elemental mapping images of TSF-KCC-1 adsorbent after Hg(II) adsorption are shown in Fig. 4. FESEM images taken after Hg(II) adsorption (Fig. 4b,c) reveal no significant difference compared to the adsorbent before Hg(II) adsorption (Fig. 2d,e). This indicates that mercury adsorption has not to effect on adsorbent morphology. Also, the distribution of structural elements (Si, O, C, and S) on the adsorbent surface is shown in Fig. 4 (second row), and the presence of mercury on the adsorbent surface after the adsorption process can be well observed. This indicates the successful adsorption of Hg(II) by the TSF-KCC-1.

### Adsorption studies

For the investigation of the applicability of the TSF-KCC-1 as a potential material for adsorption of Hg(II) cations, adsorption studies were conducted and the influence of some important experimental factors affecting the removal procedure was monitored, including pH of the Hg(II) solution, TSF-KCC-1 adsorbent dosage (*W*, mg), initial metal concentration (*C*_0_, mg L^–1^), and contact time (*t*, min). Furthermore, different kinetic and isotherm equations were used to investigate the adsorption mechanism of Hg(II) and the removal behavior of TSF-KCC-1 adsorbent material.

### Effect of solution pH and adsorbent dose on the adsorption of Hg(II) onto TSF-KCC-1 adsorbent

**Figure 5:**
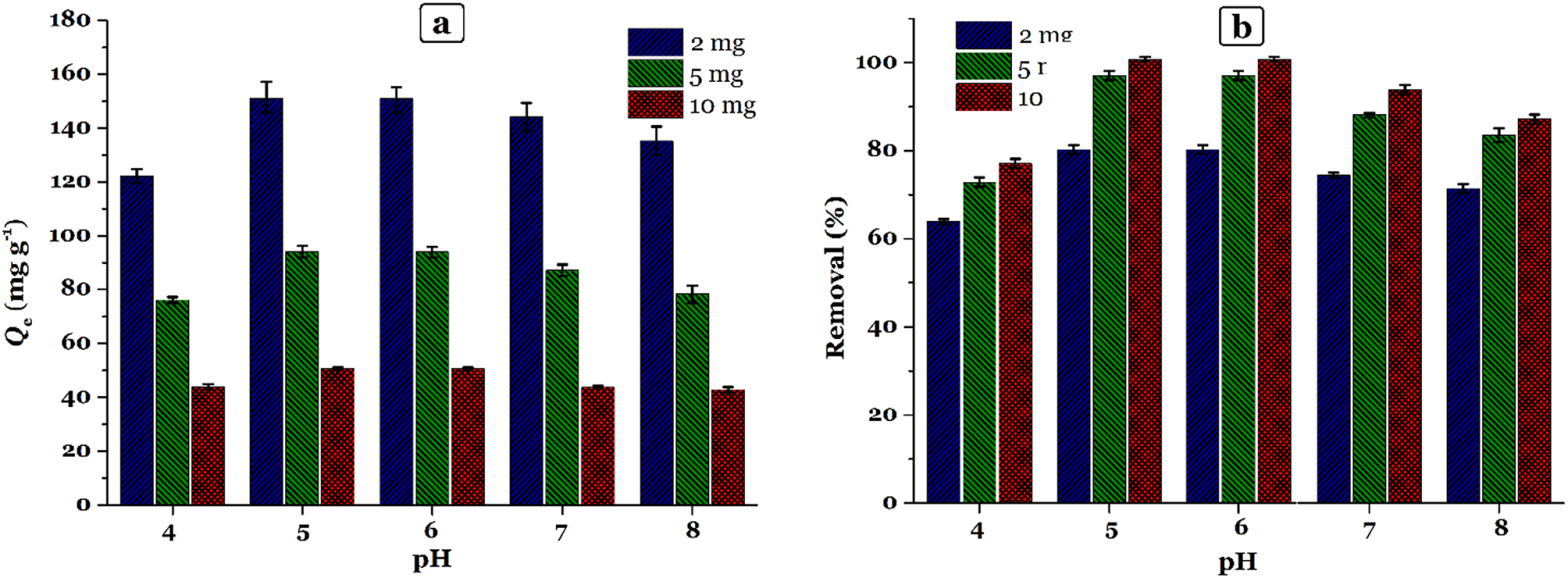
Effect of pH on the removal efficiency of Hg(II) by TSF-KCC-1 adsorbent.

Figure 5 shows the effect of the initial pH of the metal solution at different adsorbent dosages (*W* = 2, 5, and 10 mg) on the uptake of Hg(II) onto TSF-KCC-1 adsorbent at various pHs (*V* = 20 mL, *C*_0_ = 25 mg L^–1^, *T* = 25 °C, *t* = 120 min, shaking speed = 180 rpm). The obtained results represented that the adsorption of Hg(II) onto the adsorbent is a pH-dependent phenomenon. Adsorption data showed that both adsorption capacity and removal percentage increase from pH = 4 to pH = 5 and reach their maximum, and then decrease gradually and steadily as the pH increases again for all three adsorbent dosages. Maximum Hg(II) adsorption occurs at pH 5.0 as shown in Fig. 5. In this pH, there is a strong electrostatic attraction between the TSF-KCC-1 adsorbent and the Hg(II) cations, and most interactions occur between the positively charged surface of TSF-KCC-1 and adsorbate cations. Soltani and his colleagues^33–35^ have called this adsorption behavior an increasing-maximum-decreasing (IMD) pattern that occurs in the adsorption processes of heavy metal cations from aqueous media in which there is adsorbent with active functional organic groups.

A similar procedure for the adsorption of Hg(II) cations by a silica-based adsorbent was reported in previous reports^36–38^. The lower adsorption capacity at low pH (pH < 5.0) is probably due to the protonation of sulfur atoms, proton competitiveness for binding sites, in tetrasulfide groups (–S–S–S–S–) diminished the capability of complex formation with Hg(II) species. With a gradual increase in pH (pH 4.0–5.0), the concentration of H_3_O^+^ cations decreases and there is more opportunity for effective interaction of Hg(II) and tetrasulfide groups, which is observed as an increase in the amount of adsorption capacity as well as removal percentage. Then, with a further increase in the concentration of hydroxide ions (OH^–^), i.e., an increase in pH, the tendency of mercury cations to interact with sulfur groups decreases because of precipitation of solid metal hydroxide and reduced solubility, which leads to a decrease in the amount of adsorption.

Also, the adsorption capacity and removal percentage of Hg(II) by TSF-KCC-1 decreased and increased, respectively, with increasing adsorbent dosage from 2.0 to 10.0 mg. Consequently, pH 5.0 and adsorbent dosage 2.0 mg were chosen as the optimal pH and adsorbent dosage for further investigation.

### Adsorption isotherm

To investigate the isotherms of adsorption, two famous two-parameter isotherm equations, namely Freundlich and Langmuir isotherms, and a three-parameter isotherm equation, namely the Redlich-Peterson equation, are utilized and tested to fit the experimental data. The nonlinear form of the Langmuir equation can be represented as^10^

1 $${Q_e} = \, \left( {{Q_m}{_{\cdot cal}}\cdot{K_L}\cdot{C_e}} \right) \, / \, \left( {1 \, + {K_L}\cdot{C_e}} \right),$$

where *K*_L_ and *Q*_m_._cal_ are, respectively, the Langmuir isotherm constant corresponding to the energy of adsorption (L mg^–1^) and the calculated (theoretical) maximum capacity of adsorption related to the complete monomolecular layer coverage on the surface (mg g^–1^).

The Freundlich model is defined by equation^9^

2 $${Q_e} = {K_F}\cdot{C_e}^{1/n},$$

where *n* and *K*_F_ are somehow representative of the intensity and adsorption capacity (mg g^–1^) of the adsorption phenomenon, respectively. The bigness of the 1/*n* parameter represents the favorability of the uptake procedure.

The Redlich–Peterson isotherm incorporates the adsorption features of two typical Freundlich and Langmuir models into a single isotherm equation. Redlich-Peterson isotherm is defined by the equation

3 $${Q_e} = \, \left( {{K_{RP}}{C_e}} \right) \, / \, (1 \, + {a_{RP}}{C_e}^g)$$

where *K*_RP_ (Lg^–1^) and α_RP_ (mg L^–1^) ^–g^ are Redlich–Peterson isotherm constants. In the Redlich-Peterson equation *g* parameter lies between zero and unity—for *g* = 0 and *g* = 1 this equation reduces to Henri’s law and Langmuir model, respectively.

**Figure 6:**
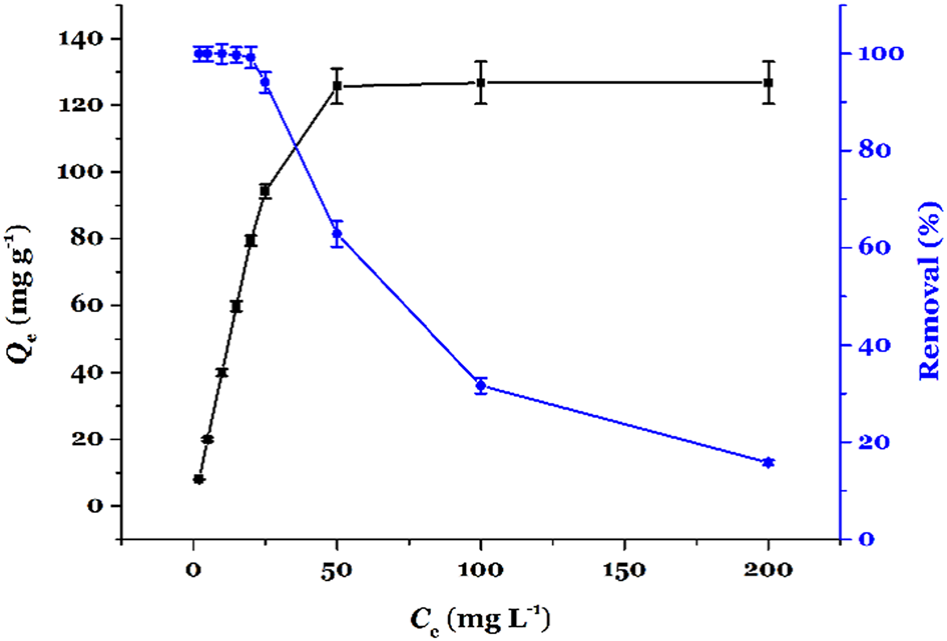
Equilibrium isotherm for adsorption of Hg(II) onto TSF-KCC-1 (■ plots are adsorption capacity and ● plots are removal percentage versus equilibrium concentration, respectively).

**Figure 7:**
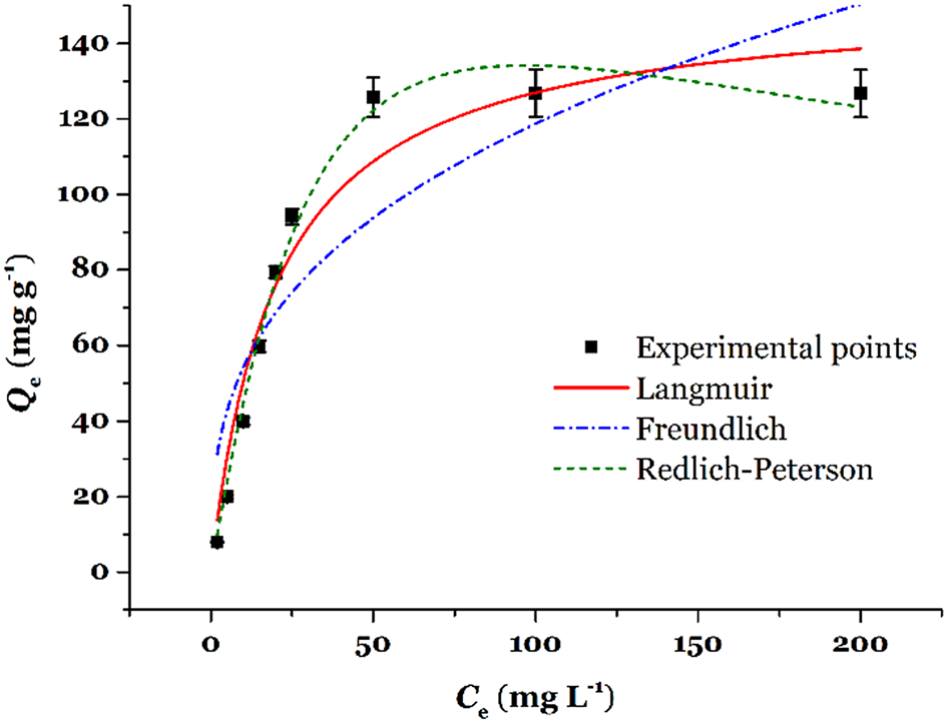
Equilibrium isotherm and non-linear fitting of isotherm models (Langmuir, Freundlich, and Redlich-Peterson).

The plots for *Q*_t_ against *C*_e_ as well as %Removal against *C*_e_ for adsorption of Hg(II) onto TSF-KCC-1 are shown in Fig. 6. The Langmuir, Freundlich, and Redlich–Peterson isotherms after nonlinear fitting are shown in Fig. 7 and R^2^ values, and their parameters are tabulated in Table 1.

**Table 1:** Isotherm parameters and their values (pH = 5.0, *V* = 20 mL, *W* = 2.0 mg, *t* = 120 min, temperature = 25 °C, shaking speed = 180 rpm).

Models	Parameters	Value
	*Q*_m,exp_/mg g^–1^	126.73
Langmuir	*R*^2^	0.9462
	*Q*_m,cal_/mg g^–1^	132.55
	*K*_L_/L mg^–1^	0.0108
Freundlich	*R*^2^	0.7731
	*n*_F_	2.93
	*K*_F_/(mg g^–1^)(mg L^–1^) ^–1/n^	24.68
Redlich-Peterson	*R*^2^	0.9877
	α_RP_/(mg L^–1^)^–g^	0.0046
	*g*/dimensionless	1.1859
	*K*_RP_/L g^–1^	4.985

As can be seen from Fig. 6, as the concentration of the Hg(II) increases, the amount of adsorption capacity and Removal percentage increases and decreases continuously, respectively. The adsorption capacity reaches its maximum value (*Q*_m,exp_ = 126.73) at an equilibrium concentration of 50 mg L^–1^. According to the *R*^2^ values, Table 1, the values of the *R*^2^ obtained from the Langmuir isotherm is larger than that of Freundlich, and calculated maximum adsorption capacity (*Q*_m, cal_ = 132.55 mg g^–1^) obtained from the Langmuir model exhibited a closer agreement with the experimental maximum adsorption capacity (*Q*_m,exp_ = 129.73 mg g^–1^). Therefore, the absorption data are better fitted with the Langmuir isotherm than the Freundlich isotherm. This may be because of the homogenous distribution of organic functional groups as surface adsorption sites on the TSF-KCC-1 adsorbent. Besides, it can be proposed that, based on the Langmuir model, there are a finite number of localized adsorption sites on the TSF-KCC-1 which are saturated after reaching the equilibrium. It was observed that the *g* value, in the Redlich-Peterson isotherm, was close to unity (*g* = 1.1859), demonstrating that the Langmuir model is more suitable than the Freundlich model for describing the mechanism of Hg(II) removal by TSF-KCC-1. This was in close agreement with the *R*^2^ values given for both Freundlich (*R*^2^ = 0.7731) and Langmuir (*R*^2^ = 0.9462) isotherm models in Table 1.

**Figure 8:**
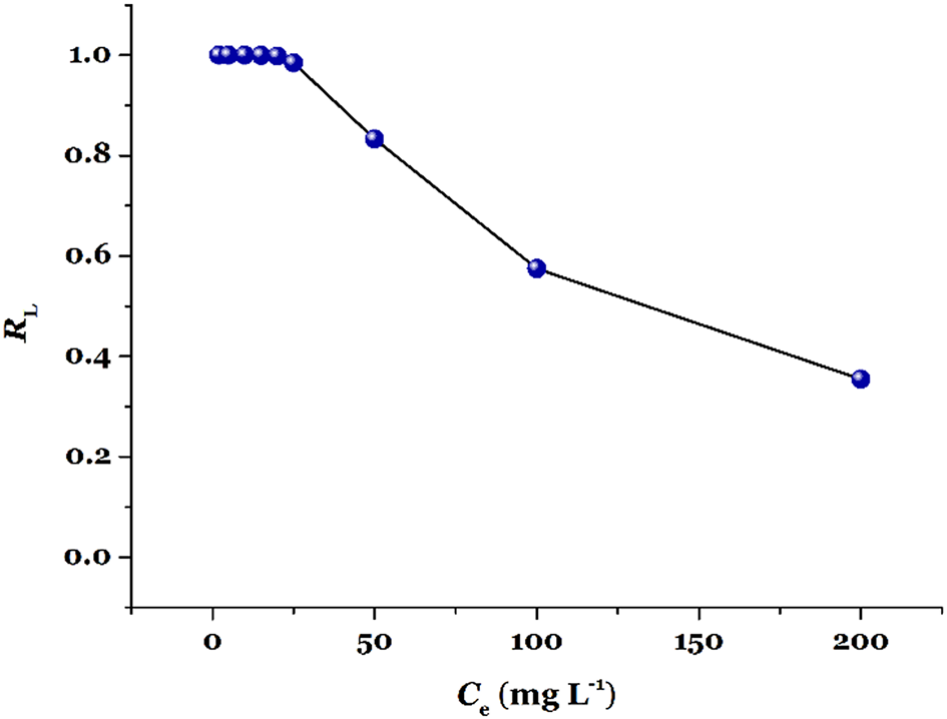
The *R*_L_ values for adsorption of Hg(II) onto TSF-KCC-1.

The so-called equilibrium parameter (*R*_L_=1/[1+(*K*_L_·*C*_e_)].) is a unitless parameter derived from the Langmuir equation which can propose the quality of an adsorption process: *R*_L_=0, irreversible, *R*_L_=1, linear; 0<*R*_L_< 1, favorable; *R*_L_>1, unfavorable adsorption process. Fig. 8 shows the *R*_L_ values against initial Hg(II) concentrations for removal of Hg(II) by TSF-KCC-1. The values obtained for *R*_L_ are between zero and unity, suggesting a very high adsorption affinity of Hg(II) cations towards TSF-KCC-1 adsorbent (favorable absorption).

### Adsorption kinetics

**Figure 9:**
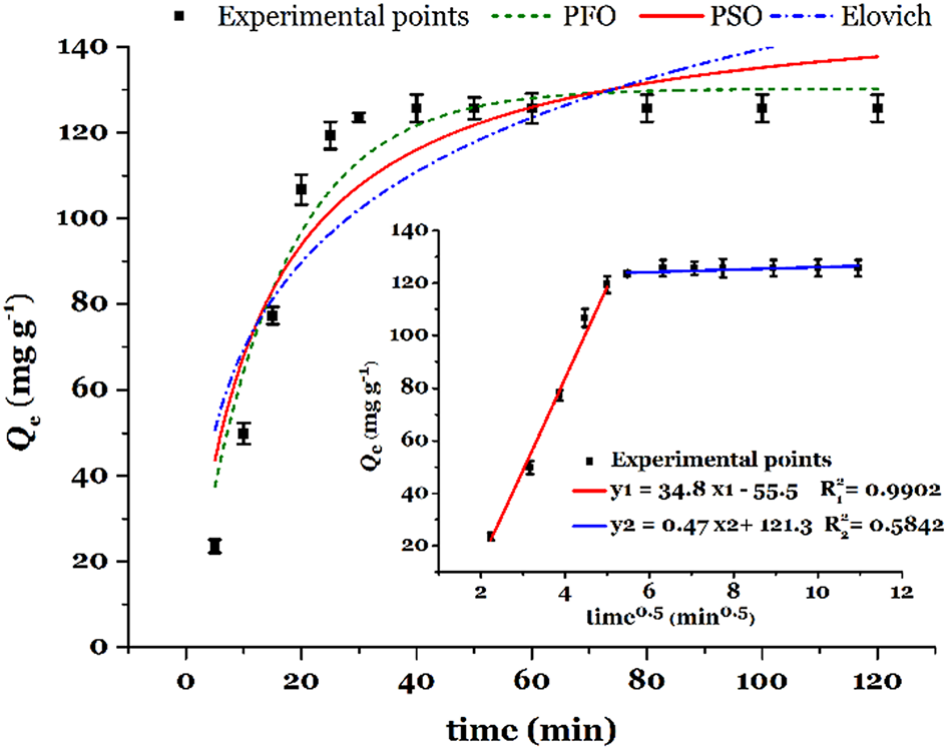
The effect of contact time on the adsorption performance of TSF-KCC-1 for Hg(II) removal and non-linear fitting of three different kinetic models (PFO, PSO, and Elovich). The inset is the IPD model.

The contact time required for achieving the steady-state uptake capacity or maximum uptake capacity of the Hg(II), is typically demonstrated as the equilibrium time. The shorter the equilibrium time, the more favorable the absorption process. In this work, the time required to reach adsorption equilibrium was found to be 40 min. (Fig. 9). This equilibrium time could be considered an acceptable amount for the removal of mercury ions from the aqueous medium.

In order to investigate the adsorption kinetics and possible adsorption mechanisms in the process of mercury adsorption by TSF-KCC-1, different kinetic models including Elovich, pseudo-first-order (PFO), and pseudo-second-order (PSO) models were used. Figure 9 shows the curves of these kinetic models after nonlinear fitting, and the obtained kinetic parameters are given in Table 2. These kinetic models are defined by the following equations^9^:

4 $$ PFO:{Q_t} = {Q_{e,cal}}(1  -  {e^{-k1 \cdot t}})$$

5 $$PSO:{Q_t} = \, ({Q_{e,cal}}^2 \cdot {k_2} \cdot t) \, / \, (1 \, + {Q_{e,cal}} \cdot {k_2} \cdot t)$$

6 $$Elovich: Q_{t} {\text{ }} = \left( {1/\beta } \right)\ln \left( {\alpha /\beta } \right)t $$

where *Q*_e,cal_, *k*_1_, *k*_2_, are adsorption capacity at equilibrium (mg g^–1^), PFO rate constant (min^–1^), PSO rate constant (g mg^–1^ min^–1^), respectively. a and b represent the Elovich initial adsorption rate (mg g^–1^ min^–1^) and the Elovich constant (g mg^–1^), respectively.

**Table 2:** Kinetics adsorption parameters (pH = 5.0, *C*_0_ = 50 mg^–1^ L, *V* = mL, *W* = mg, temperature = 25 °C, shaking speed = 180 rpm).

Models	Parameters	Value
	*Q*_e,exp_/mg g^–1^	125.68
PFO	*R*^2^	0.9281
	*Q*_e,cal_/mg g^–1^	130.27
	*k*_1_/min^–1^	0.068
PSO	*R*^2^	0.8473
	*Q*_e,cal_/mg g^–1^	151.98
	*k*_2_/g mg^–1^ min^–1^	5.3 × 10^–4^
Elovich	*R*^2^	0.7302
	α/mg g min	24.884
	β/ g mg^–1^	0.0313
IPD	*R*_1_^2^	0.9902
	*k*_IPD,1_	34.79
	*C*_1_	–55.3
	*R*_2_^2^	0.5842
	*k*_IPD,2_	0.4725
	*C*_2_	0.5842

According to Table 2, the *R*^2^ values obtained after the nonlinear fitting analysis indicate that the PFO model (*R*^2^ = 0.9281) fits the experimental data much more accurately than the PSO (*R*^2^ = 0.8473) and Elovich (*R*^2^ = 0.7302) kinetic models. Also, compared to the PSO model (*Q*_e,cal._ = 151.98 mg g^–1^), the PFO model has a closer calculated absorption capacity (*Q*_e,cal._ = 130.27 mg g^–1^) to the experimental absorption capacity (*Q*_e,exp._ = 125.68 mg g^–1^), which indicates a very good agreement of the PFO model with the experimental data.

The Intraparticle diffusion (IPD) kinetic equation is applied to ascertain the rate-limiting step (RLS) in the uptake process as well as to determine the number of possible steps in the adsorption process. This kinetic model is defined by the following equation^27^:

7 $$ IPD:{Q_t} = {k_{IPD}}{t^{0.5}} + C$$

where *k*_dif_ and *C* are the IPD rate constant (mg g^–1^ min^–0.5^) and IPD constant related to the thickness of the boundary layer (mg g^–1^), respectively. The plots of *Q*_t_ (mg g^–1^) versus *t*^0.5^ (min^0.5^) are revealed in Fig. 8 (inset) and the values of IPD parameters are given in Table 2. In the IPD model, if the graph (*Q*_t_ versus *t*^0.5^) is linear that passes through the origin (*C* = 0), it means that the absorption follows the intraparticle diffusion mechanism, and if the graph is multilinear that does not pass through the origin, it means that the adsorption consists of several stages and some degree of boundary layer affect the removal procedure. As shown in Fig. 9 (input), the IPD diagram consists of two separate steps. The first step has a higher linear slope which is attributed to external mass transfer effects or external surface adsorption (boundary layer diffusion) and the second step has a lower slope which is related to the gradual adsorption phenomenon with control of the IPD mechanism (RLS)^8^. Also, the deviation of the IPD diagram from the origin indicates that in the process of Hg(II) adsorption by TSF-KCC-1, the IPD model can not explain the adsorption mechanism alone, but other mechanisms (PFO model) also affect the adsorption.

### Regeneration of TSF-KCC-1

**Figure 10:**
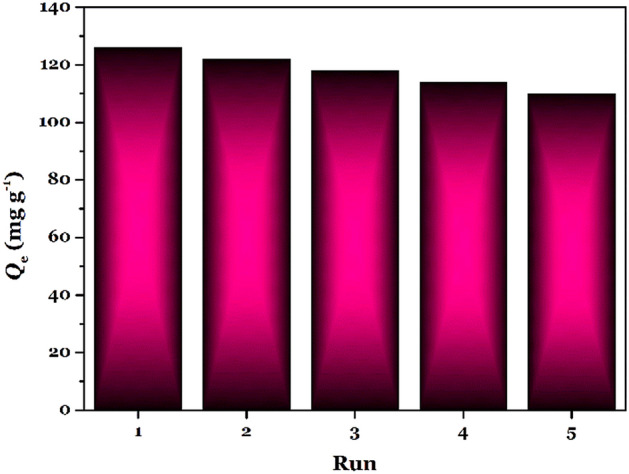
Regeneration of TSF-KCC-1.

The economic viability of any adsorption procedure directly depends upon the adsorbent regeneration ability for several cycles of the adsorption procedure. In this work, a regeneration investigation was carried out to assess the reuse potential of TSF-KCC-1 (adsorption conditions: pH = 5.0, *V* = 20 mL, *W* = 2.0 mg, *t* = 120 min, temperature = 25 °C, shaking speed = 180 rpm). 0.2 mol L^–1^ HCl was utilized as an eluent solution to desorb the Hg(II) cations from the TSF-KCC-1. To investigate the adsorption potential of regenerated TSF-KCC-1, five consecutive cycles of Hg(II) adsorption–desorption studies were conducted and the results are presented in Fig. 10. The adsorption–desorption result demonstrated that TSF-KCC-1 possesses good reuse potential and only 16 mg g^–1^ reduction in Hg(II) adsorption capacity was monitored at the end of the five consecutive cycles. The adsorption–desorption results obviously reveal that TSF-KCC-1 is a potential candidate for the adsorptive removal of Hg(II) cations from water.

### Adsorption mechanism

Based on the isothermal and kinetic data obtained, the proposed adsorption mechanism for Hg(II) adsorption by TS-KCC-1 includes a rapid (PFO model) monolayer adsorption (Langmuir model) consists of two general steps (IPD): 1) The first step is attributed to boundary layer diffusion, external mass transfer effects or external surface adsorption, and 2) the second step is related to the gradual adsorption phenomenon with control of the IPD mechanism.

### Comparison study

The synthesized TS-KCC-1 showed a considerably enhanced adsorption capacity toward Hg(II) compared with most adsorbents reported in Table 3. Among the adsorbents reported in Table 3, only the adsorbent synthesized by Soltani and his colleagues has a higher adsorption capacity than TS-KCC-1. Nevertheless, this adsorbent can still be used as an effective adsorbent for the removal of mercury ions in aqueous media. The reason for the good adsorption capacity of this adsorbent compared to most of the adsorbents reported in Table 3 is probably due to the unique fibrous structure of the TS-KCC-1 and the availability of its adsorption sites on its fibers.

**Table 3:** The maximum adsorption capacities for Hg(II) reported in previous works.

Adsorbent^*a*^	Research group	Year	*Q*_e,cal._/mg g^–1^	References
TS-KCC-1	Marjani et al	2021	132.55	This work
NiCo-LDH/MOF NC	Soltani et al	2021	509.8	^39^
α-Fe_2_O_3_	Zhang et al	2018	11.16	^40^
SMs BTESPT-SMs MPTMS-SMs	Saman et al	2014	20.0 37.0 62.3	^41^
GG-based adsorbent	Thakur et al	2014	41.13	^42^
Starch-based adsorbent	Huang et al	2011	131.2	^43^
FTU-SBA-15	Mureseanu et al	2010	122.4	^44^
MBT-MCM-41 MBT-SBA-15	Pérez-Quintanilla et al	2006	42.1 48.1	^45^
MTZ-MCM-41	Pérez-Quintanilla et al	2006	140.4	^46^
MP-MCM-41 MP-SBA-15	Pérez-Quintanilla et al	2006	38.1 20.0	^47^

^a^*NiCo-LDH/MOF NC* functionalized Ni_50_Co_50_-layered double hydroxide/UiO-66-(Zr)-(COOH)_2_ nanocomposite, *X-SMs* X-functionalized silica microspheres (BTESPT: bis(triethoxysilylpropyl) tetrasulfide, *MPTMS* 3-mercaptopropyl trimethoxysilane), *GG* guar gum, *FTU-SBA-15* 1-furoyl thiourea-functionalized SBA-15, *MBT-MCM-41/SBA-15* 2-mercaptobenzothiazol-functionalized MCM-41/SBA-15, *MTZ-MCM-41* 2-mercaptothiazoline-functionalized MCM-41, *MP-MCM-41/SBA-15* 2-mercaptopyridine-functionalized MCM-41/SBA-15.

## Conclusion

For the first time, TS-F-KCC-1 was synthesized by a simple, conventional ultrasonic-assisted, sol–gel-hydrothermal synthesis approach. This work has demonstrated the great potential of a simple, conventional ultrasonic-assisted, sol–gel-hydrothermal synthesis approach. for the formation of homogeneously distributed mesoporous TS-F-KCC-1 and its excellent adsorption capability for Hg(II). Adsorptive removal of Hg(II) onto TSF-KCC-1 in a batch system has been carried out. Isotherm results agreed very well with the Langmuir model with a maximum adsorption capacity of 132.55 mg g^–1^ at constant conditions (pH: 5.0 for; adsorbent dose: 5.0 mg; the volume of the solution: 20.0 mL; time: 40 min; temperature: 198 K; shaking speed 180 rpm). Among different kinetic models, the PFO equation was better fitted since experimental data agreed very well with theoretical data. TS-KCC-1was shown to be a promising functional nanoporous material for the uptake of Hg(II) cations from aqueous media. To the best of our knowledge, there has been no report on the uptake of toxic Hg(II) cations by TS-F-KCC-1 prepared by a conventional ultrasonic-assisted sol–gel-hydrothermal synthesis method. It is hoped that this adsorption study will contribute to a deeper understanding of the role of the fabrication of functionalized fibrous silica nanospheres for the removal of hazardous heavy metals using an adsorptive removal strategy.

## Experimental section

### Materials

Cetyltrimethylammonium bromide (CTAB, ≥ 97.0%), tetraethyl orthosilicate (TEOS, ≥ 99.0%), bis[3-(triethoxysilyl)propyl] tetrasulfide (TESPT, ≥ 90.0%), urea (≥ 99.5%), cyclohexane (≥ 99.9%), 1-pentanol (≥ 98.5%), sodium hydroxide(≥ 97.0%), mercury (II) nitrate monohydrate (≥ 98.0%), hydrochloric acid (37%), ethanol (96 and 99%), acetone, and toluene (≥ 99.0%) were purchased from Merck (Darmstadt, Germany).

### Apparatus

Furrier transforms infrared (FTIR) spectra of the powder samples were recorded in KBr on a Perkin Elmer Spectrum RX-1 FT-IR spectrophotometer (Perkin–Elmer, USA) in a wavelength range 4000–400 cm^−1^. Morphology of the produced samples was determined by a Field Emission-Scanning Electron Microscope (FESEM, MIRA3 TESCAN-XMU, Kohoutovice, Czech Republic) equipped with energy dispersive spectroscopy (EDS). Transmission Electron Microscopic (TEM) images of the samples were viewed in a Philips CM120 Electron Microscope (Eindhoven, The Netherlands) operating at 120 kV. Also, the concentrations of Hg(II) cations in aqueous media were measured using atomic absorption (AA) spectrophotometer (PerkinElmer PinAAcle™ 900 T, Shelton, CT, USA) equipped with a mercury hollow cathode lamp and with a detection limit of 0.3 mg L^–1^.

### Synthesis of fibrous silica KCC-1 and tetrasulfide-functionalized KCC-1 (TSF-KCC-1)

Pristine KCC-1 was prepared via the facile sol–gel hydrothermal-assisted process according to the method described by Soltani and co-workers^11,14^ with some modifications. In a 1000-mL Teflon container, a mixture of pure water (250.0 mL), CTAB (2.500 g), and urea (2.400 g) were vigorously stirred at 25 °C for 20 min. Then, a mixture of TEOS (12.500 g) in cyclohexane (250.0 mL) was gently added to the above mixture under vigorous stirring at 25 °C until a homogeneous milky solution was obtained. Afterward, 1-pentanol (15.0 mL) was slowly added to the Teflon container under vigorous stirring over 5 min at 25 °C. The resulting milky solution was further vigorously stirred for 30 min at 25 °C. The Teflon container was sealed in a stainless steel autoclave and placed in a preheated electric oven (120 °C). After hydrothermal treatment (6 h), the autoclave was allowed to cool down to ambient temperature, and a white gel-like product was isolated by centrifugation (6000 rpm), rinsed several times with water and ethanol, air-dried at 70 °C overnight, and furnace-calcined at 550 °C for 6 h under air atmosphere to remove organic template.

### Synthesis of tetrasulfide-functionalized KCC-1 (TSF-KCC-1)

Post-modification of KCC-1 with TESPT was carious out according to a simple g protocol reported by Soltani and co-workers^11,14^. 2.00 g of KCC-1 and 250 mL dry toluene were added in a 500-mL round bottom flask and ultrasonicated for 15 min to deagglomeration of particles. Afterward, a certain amount of TESPT was added to the flask, and the resulting mixture was magnetically stirred under reflux in an oil bath under a nitrogen atmosphere overnight. Finally, the reaction mixture was allowed to cool down to ambient temperature, and the white product was separated by centrifugation (6000 rpm), rinsed several times with toluene and ethanol to eliminate unreacted TESPT molecules, and oven-dried at 70 °C for a day to get fine white particles, abbreviated as TSF-KCC-1.

### Adsorption experiments

Adsorption experiments were carried out in 50-mL stoppered polyethylene (PP) bottles. For this purpose, a certain amount of TSF-KCC-1 adsorbent (2, 5, and 10 mg) was mixed with 20.0 mL of Hg(II) solutions of 2–200 mg L^–1^ concentration range at different pH conditions (pH 4.0, 5.0, 6.0, 7.0, and 8.0). The bottles with their contents were then shaken mechanically for 120 min at 25 °C. Finally, the solutions were centrifuged for 5 min at 4000 rpm, and the residual Hg(II) concentrations in the solutions were measured employing an AA spectrophotometer. The removal percentage of Hg(II) from aqueous media at any time *t* and equilibrium are calculated by %Removal = 100·[(*C*_i_ – *C*_t_)/*C*_i_] and %Removal = 100·[(*C*_i_ – *C*_e_)/*C*_i_], respectively, where *C*_i_, *C*_t_, and *C*_e_ are the initial, time-dependent, and equilibrium concentrations (mg L^–1^) of Hg(II) cations in aqueous media, respectively. The adsorption capacities of the adsorbent for Hg(II)—in milligrams of Hg(II) per gram of TSF-KCC-1 adsorbent—at any time *t* (*Q*_t_) and at equilibrium (*Q*_e_) are calculated by *Q*_t_ = (*V*/*W*) · ( *C*_i_ – *C*_t_) and *Q*_e_ = (*V*/*W*) · ( *C*_i_ – *C*_e_), respectively, where *V* and *W* are, respectively, the volume of the aqueous solution (L or mL) and mass of the adsorbent (g or mg). All the kinetic and isotherm models were fitted by the non-linear regression analysis (NLRA) employing the statistical analysis function in Origin Origin Pro 9.0 (Origin Lab Corporation, Northampton, USA).
